# Rapid sequence spinal anesthesia for the most urgent cesarean section: a simulation and clinical application

**DOI:** 10.1186/s40981-016-0037-6

**Published:** 2016-06-02

**Authors:** Kotaro Hori, Yutaka Oda, Masayoshi Ryokai, Ryu Okutani

**Affiliations:** Department of Anesthesiology, Osaka City General Hospital, 2-13-22 Miyakojima-hondori, Miyakojima-ku, Osaka, 534-0021 Japan

**Keywords:** Cesarean section, Rapid sequence spinal anesthesia, Simulation

## Abstract

Rapid sequence spinal anesthesia is a recently developed technique for the most urgent, category-1 cesarean section. To successfully perform this technique, it is important to multi-disciplinarily discuss with all staffs related to delivery, make a local protocol in each hospital and simulate the procedure with them. Owing to the above preparation, we were able to perform the technique smoothly also in the real patient. Considering possible benefits of rapid sequence spinal anesthesia, we should prepare enough before we use it in the actual clinical situations.

## Correspondence/Findings

To the Editor:

Rapid sequence spinal anesthesia (RSS) is a recently developed technique for the most urgent cesarean section, category-1 in the National Institute for Clinical Excellence (NICE) guideline, where general anesthesia has extensively been performed [1–3]. In terms of safety of anesthesia, if we do not have to think about time constraints, spinal anesthesia is basically safer, and RSS is designed to satisfy also the time constraints. Different from spinal anesthesia for elective cesarean section, RSS is characterized by specific anesthetic procedure including the methods of sterilization, dose of anesthetics, required level of spinal anesthesia before starting surgery for shortening the decision-delivery interval [1, 2]. However, it is important to note that successful RSS requires effective deployment of medical staffs and teamwork, as suggested by Kinsella [1]. We recently created a local protocol of RSS in our hospital after multi-disciplinarily discussion, and performed its simulation, which significantly contributed to perform RSS successfully for category-1 cesarean section.

After making a decision to introduce RSS in our hospital, we initially informed obstetricians, pediatricians, nurses in the operating room, obstetric suite and neonatal intensive care unit of the details of RSS, and held repeated discussion to clarify the role of each staff to create a local protocol of RSS. This protocol includes the role of each staff for postural change and measuring vital signs, dose for spinal anesthesia (if possible, add fentanyl 25 μg, and if not, increased 0.5 % bupivacaine at a maximum dose of 3.0 ml), and T10 cold sense block to start surgery. Simulation of RSS by all the related staffs was also performed, which was beneficial for all members to understand the differences between RSS and spinal anesthesia for elective cesarean section and to recognize their roles in the practice of RSS.

Following creation of the protocol and simulation of RSS, we performed RSS in a parturient, who was admitted to our hospital at 36 weeks’ gestation with a history of suprapubic pain. The fetal heart rate progressed to a persistent bradycardia and category-1 cesarean section was determined. After brief discussion with an obstetrician, we decided to perform RSS. She was immediately taken to the operating room, and transferred to surgical bed in the right lateral position. Following skin preparations, 2.0 ml hyperbaric bupivacaine 0.5 % and 25 μg fentanyl was injected through L 3/4, and she was positioned to supine. After confirming loss of cold sensation at T10 [1, 2], cesarean section was started. The baby was delivered with the Apgar score of 8/9 at 1/5 min. The decision-delivery interval was 20 min, which was similar to a case series from United Kingdom, where RSS was first reported (22.5 ± 5.9 min; mean ± SD) [1]. Many hospitals do not have the local protocol of RSS even in the United Kingdom [4], much less in Japan. Considering possible benefits of rapid sequence spinal anesthesia, we should prepare enough before we use it in the actual clinical situations.

## Ethics approval and consent to participate

Not applicable.

## Consent for publication

Written informed consent was obtained from the patient for publication of this letter. A copy of the written consent is available for review by the Editor-in-Chief of this journal.

## Abbreviations

NICE: National Institute for Clinical Excellence
RSS: rapid sequence spinal anesthesia
